# Immune Cell Infiltration-Based Characterization of Triple-Negative Breast Cancer Predicts Prognosis and Chemotherapy Response Markers

**DOI:** 10.3389/fgene.2021.616469

**Published:** 2021-03-19

**Authors:** Yufei Lv, Dongxu Lv, Xiaohong Lv, Ping Xing, Jianguo Zhang, Yafang Zhang

**Affiliations:** ^1^Department of Anatomy, Harbin Medical University, Harbin, China; ^2^College of Bioinformatics Science and Technology, Harbin Medical University, Harbin, China; ^3^Department of Ultrasound, The First Affiliated Hospital of Harbin Medical University, Harbin, China; ^4^Department of Breast Surgery, The Second Affiliated Hospital of Harbin Medical University, Harbin, China

**Keywords:** triple negative breast cancer, tumor microenvironment, survival biomarkers, chemotherapy-response, bioinformatics

## Abstract

Breast cancer represents the number one cause of cancer-associated mortality globally. The most aggressive molecular subtype is triple negative breast cancer (TNBC), of which limited therapeutic options are available. It is well known that breast cancer prognosis and tumor sensitivity toward immunotherapy are dictated by the tumor microenvironment. Breast cancer gene expression profiles were extracted from the METABRIC dataset and two TNBC clusters displaying unique immune features were identified. Activated immune cells formed a large proportion of cells in the high infiltration cluster, which correlated to a good prognosis. Differentially expressed genes (DEGs) extracted between two heterogeneous subtypes were used to further explore the underlying immune mechanism and to identify prognostic biomarkers. Functional enrichment analysis revealed that the DEGs were predominately related to some processes involved in activation and regulation of innate immune signaling. Using network analysis, we identified two modules in which genes were selected for further prognostic investigation. Validation by independent datasets revealed that CXCL9 and CXCL13 were good prognostic biomarkers for TNBC. We also performed comparisons between the above two genes and immune markers (CYT, APM, TILs, and TIS), as well as cell checkpoint marker expressions, and found a statistically significant correlation between them in both METABRIC and TCGA datasets. The potential of CXCL9 and CXCL13 to predict chemotherapy sensitivity was also evaluated. We found that the CXCL9 and CXCL13 were good predictors for chemotherapy and their expressions were higher in chemotherapy-responsive patients in contrast to those who were not responsive. In brief, immune infiltrate characterization on TNBC revealed heterogeneous subtypes with unique immune features allowed for the identification of informative and reliable characteristics representative of the local immune tumor microenvironment and were potential candidates to guide the management of TNBC patients.

## Introduction

11.6% of all cancers in women were found to be breast cancer, making it the most commonly diagnosed malignancy in this population as well as the leading cause of cancer death (Bray et al., 2018). Approximately 15% of all breast cancers are triple-negative breast cancers (TNBC), which do not express ER, PR, or HER2 (Fallahpour et al., 2017). Due to its genetic profile, TNBC is often insensitive to anti-hormonal therapy or monoclonal antibodies (Li et al., 2018; Buiga et al., 2019; Ma et al., 2020). Chemotherapy remains the main treatment modality for TNBC patients, which confers controversial outcomes on patients despite some literature touting its benefits (Pomponio et al., 2019; Steenbruggen et al., 2020). TNBC therefore is synonymous with a poor outcome in breast cancer in contrast to other subtypes, and is generally regarded as the most aggressive breast cancer subtype.

Therefore, more efficient prognostic and therapeutic strategies for TNBC are still urgently needed. Increasing evidence highlights the critical role of the immune system in the initiation and progression of cancers (Wang et al., 2018; Cai et al., 2019; Tu et al., 2020). TNBC is associated with a high density of tumor-infiltrating lymphocytes (TILs) defined by histopathology evaluation, which represents a robust intratumoral inflammatory response describing triple negative tumors as an immunogenic neoplasia (Nederlof et al., 2019; Romero-Cordoba et al., 2019). In addition to TIL count by immune pathological evaluation, other methods recently emerged to assess the tumor immune landscape such as deconvolution algorithm to define the proportion of immune cells using genomic profiling (Loi et al., 2019), to identify the gene expression signatures that distinguish the immune-state, and then to be a potential prognostic factor (Romero-Cordoba et al., 2019). Immune checkpoint molecules are able to inhibit or activate the immune system. The expression or functional enhancement of inhibitory immune components (PD-1, CTLA-4, TIM-3, etc.) weakens the immune system and increases susceptibility to cancers (Tuccilli et al., 2018; Moore et al., 2019; Salgado et al., 2020). Recent research has demonstrated that TNBC possesses higher immunogenicity than other subtypes (Denkert et al., 2018). PD-1 and PD-L1 were highly expressed in TNBC in contrast to other subtypes, indicating that the patients benefit more from immune therapies (Zhou et al., 2018). It is widely accepted that TNBC patients respond well to immune checkpoint inhibitors such as PD-1 inhibitors (Nanda et al., 2016; Voorwerk et al., 2019). Nevertheless, PD-1 inhibitor sensitivity is not universal amongst all TNBC samples given the irregular expression of PD-1 and PD-L1 (Barrett et al., 2018). This challenges the current genomic-based breast cancer classification. Further delineation of comprehensive immunological signature patterns may serve to develop an immunological-based classification strategy and prime the field toward personalized therapy (Kawashima et al., 2018; Li et al., 2020). Previous studies on TNBC microenvironment subtypes are heterogenous (Costa et al., 2018; Romero-Cordoba et al., 2019; Xiao et al., 2019; Chen et al., 2020). However, a comprehensive landscape of the TNBC microenvironment, its impact on therapeutic responses, and TME-related prognostic markers are still not well-characterized.

In this study, we evaluated the relative quantity of immune-microenvironment heterogeneity in TNBC tissues and its characteristics. The patients were clustered into two immune clusters based on the ssGSEA result. The prognostic significance and the potential immune related gene signatures were then characterized. Finally, we evaluated the ability of these signatures to predict patient chemotherapeutic response.

## Materials and Methods

### TNBC Datasets and Preprocessing

Publicly available TNBC gene-expression data sets and the corresponding clinical datasets were extracted from the Molecular Taxonomy of Breast Cancer International Consortium (METABRIC), The Cancer Genome Atlas (TCGA), and from the Gene Expression Omnibus (GEO) (Curtis et al., 2012). cBioPortal was used to download METABRIC and TCGA genomic data. Sample amount, baseline data, and clinical endpoints of all GDC datasets were assessed using R and R Bioconductor packages. Sample inclusion criteria were those that contained information regarding overall survival.

The independent dataset GSE12276 from the GEO database and TCGA dataset was used in the validation of univariate Cox regression of CXCL9 and CXCL13. The datasets GSE25055, GSE58812, and GSE103091 were used in the validation of multivariate Cox regression analysis.

Two cohorts (GSE18728 and GSE137356) were used to assess TNBC patient response to chemotherapy. The robust multichip average (RMA) was used to normalize affymetrix-generated raw CEL files which were downloaded from the GEO dataset.

### Calculation of Microenvironment Cell Abundance

The GSVA R package (version 1.24.0) was used to implement single sample gene set enrichment analysis (ssGSEA) (Hanzelmann et al., 2013). Enrichment scores of 782 genes representing 28 types of immune cells were calculated to determine the normalized enrichment score of 27 types of immune cells represented by 782 gene signatures which were collected from a wide range of existing literature (Newman et al., 2015; Senbabaoglu et al., 2016; Wang et al., 2020). The Cell Type Identification by Estimating Relative Subsets of Known RNA Transcripts (CIBERSORT) algorithm allowed for estimation of infiltrating immune cell composition (Newman et al., 2015). CIBERSORTX method^1^ was also used. The ESTIMATE algorithm was performed to calculate the ESTIMATE, stromal, immune, and tumor purity scores for TNBC patients in the bulk gene expression profiles (Yoshihara et al., 2013).

### Collection of Immune-Related Data

The cytotoxic activity (CYT) score for each patient was determined as an average of the PRF1 and GZMA expression levels, which were known to be closely related to CD8 + T cell activation in tumors (Rooney et al., 2015). The Tumor Inflammation Signature (TIS) score was derived from the average of log2- transformed gene expression of the determined marker genes (Ayers et al., 2017). To investigate tumor infiltrating T cells, the proportion of tumor infiltrating lymphocytes (TILs) was also calculated (Bindea et al., 2013). Relative antigen presentation machinery (APM) was calculated using a validated gene expression signature (Zhang et al., 2018).

### Differentially Expressed Genes (DEGs) Between the Tumor Immune Clusters

DEGs between high infiltration clusters and low clusters were identified using the R package limma.

DEGs were determined by significance criteria (| logFC| > 1, *P* < 0.01) as previously implemented. The adjusted *P*-value for multiple testing was quantified utilizing the Benjamini–Hochberg correction.

### Enrichment Analysis

The DEGs identified above were used for functional enrichment analyses, which were performed using the online gene annotation and analysis tool Metascape^2^ (Zhou et al., 2019), as well as the Database for Annotation, Visualization, and Integrated Discovery (DAVID) tool. Network analysis was done using Search Tool for the Retrieval of Interacting Genes (STRING^3^) online database (combined score >0.9). Cytoscape 3.7.2 was utilized to visualize the interactive network.

### Statistical Analysis

The R program (version 3.5.0) was used to carry out all statistical analyses. The single sample gene set enrichment analysis (ssGSEA), implemented in the GSVA R package (version 1.38.0), was also introduced to calculate the normalized enrichment score (NES) of TNBC samples. The limma R package (version 3.22.5) was used to analyze differentially expressed gene (DEG) for the read count data from TNBC samples. The ggplot2 (version 2.2.1) and pheatmap (version 1.0.12) were used to draw the heatmaps and other plots. The R package ggpubr (version 0.4.0) was used to plot the vinlioplot. The R package forestplot (version 1.10.1) was used to plot the forest plot.

The Kaplan-Meier method was used to gain an estimate of the survival curves. The survival differences were assessed utilizing the two-sided log-rank test. Correlation between immune factors was calculated using Pearson’s correlation coefficients. The Kaplan–Meier survival curves were visualized through the use of the survfit function in the R package survival (version 3.2–7).

## Results

### Comprehensive Immune-Cell Infiltrate Profile in Triple Negative Breast Cancer

**FIGURE 1:**
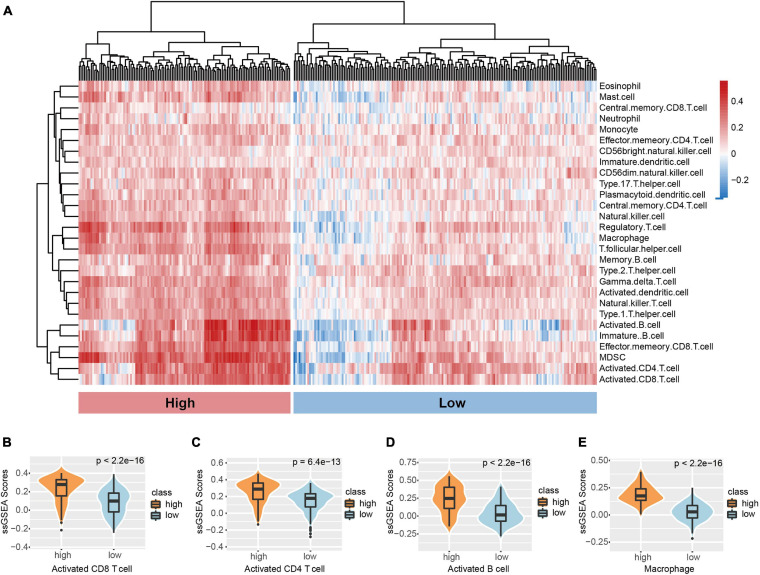
Microenvironment phenotypes of TNBC. **(A)** Hierarchical clustering of TNBC immune infiltration phenotypes based on the ssGSEA score of 28 microenvironment cell subsets. Signature scores of **(B)** CD8 T cell, **(C)** CD4 T cell, **(D)** B cell, and **(E)** Macrophage among clusters. The boxplot is depicted within the violin plot.

To assess the range and types of immune cell infiltration, the ssGSEA method was used to estimate the enrichment of 27 types of immune cells for 299 TNBC patients derived from the METABRIC dataset who had existing transcriptome and clinical features data. By applying the unsupervised hierarchical agglomerative clustering, the TNBC tumors were subsequently re-classified into two heterogeneous clusters: low infiltration (176) and high infiltration (123) (Figure 1A). We further demonstrated the infiltrate abundance of some immune cells to validate our microenvironment clustering. The group with stronger immune cell activity were noted to be more highly enriched with macrophages and B cells as well as CD8^+^ and CD4^+^ cells (Figures 1B–E); the low infiltration subtype had a relatively lower abundance of immune-active cells (Figures 1B–E). In addition, the ESTIMATE algorithm was used to obtain the stromal scores, estimate scores, and immune scores. Significant differences between the scores from the two immune clusters were observed (Supplementary Figures 1A–C). To determine if genotype-predicted immune phenotypes correlated with tumor cell immune cell infiltrate profiles, levels of APM, CYT, TIL, and TIS between two immune subtypes were evaluated (Supplementary Figures 1D–G). CYT scores were markedly higher in the high infiltrate cluster compared to the low infiltrate cluster (*p* < 2.2e−16) (Supplementary Figure 1D). Similarly, significantly higher APM, TILs, and TIS were observed in the high infiltration cluster (Supplementary Figures 1E–G).

### Prognostic Analysis of Microenvironment Phenotypes

**FIGURE 2:**
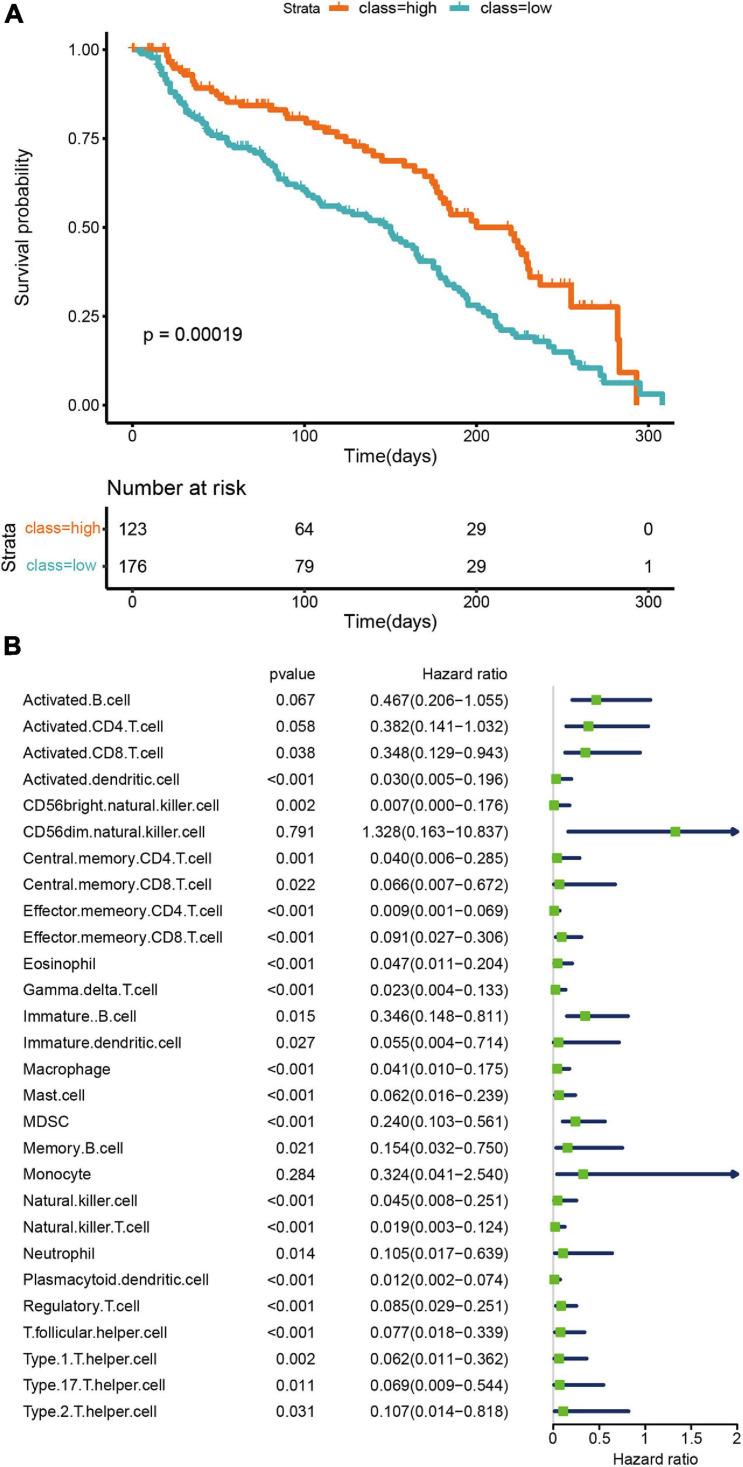
Prognostic significance of microenvironment clusters and cells in TNBC. **(A)** OS Kaplan–Meier curves of each cluster. **(B)** A univariate Cox proportional hazards model was used to estimate the prognostic value of each cell subset. For each cell type, the line length represents 95% confidence interval. HR < 1.0 represent that a cell type is a favorable prognostic biomarker.

The prognostic ability of the tumor microenvironment was also assessed in this study. Survival analyses demonstrated distinct clinical outcomes between the two TNBC subtypes. Those of the high infiltration cluster were noted to have markedly better overall survival (OS) in contrast to the low infiltration cluster (*p* = 0.00019; Figure 2A). Univariate Cox regression was conducted to analyze the relationships between 27 human immune cell phenotypes and patient outcomes. We found that the expression of several immune cell phenotypes significantly correlated to overall survival. For example, activated CD8 + T cells and natural killer cells were significantly correlated to better overall survival (CD8 + T cells: *P* = 0.038, HR = 0.348, 95% CI = 0.129−0.943); natural killer cells: *P* < 0.001, HR = 0.045, 95% CI = 0.008−0.251). At the same time, high levels of macrophages were also associated with good prognosis (macrophages: *P* < 0.001, HR = 0.041, 95% CI = 0.010−0.175).

### Differential Expressed Genes With Immune Clustering

**FIGURE 3:**
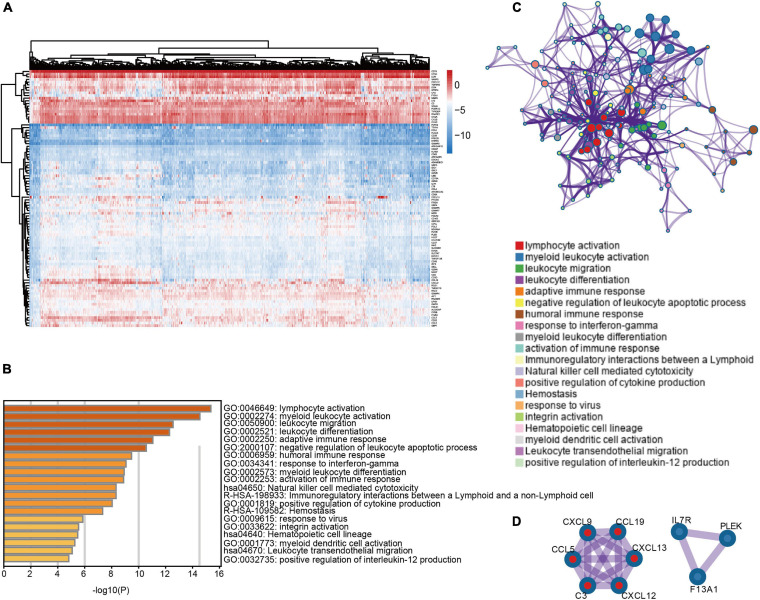
Differentially expressed genes between immune microenvironment clusters and related functional annotations **(A)** Heat map of 110 DEGs suggested distinct mRNA expression profiles in the METABRIC dataset. **(B)** Functional enrichment analysis including GO and pathways was performed using 110 DEGs. **(C)** The network of the top 20 clusters of enriched terms. **(D)** Two PPI network modules including nine genes were identified.

To explore differential expressed genes (DEGs) between high and low infiltration clusters, the limma package algorithm was performed on the METABRIC dataset. A total of 110 genes, including 31 downregulated and 79 upregulated genes, were found in the high infiltration cluster. Cluster analysis and heatmap including 110 DEGs are shown in Figure 3A. Functional enrichment analysis, including GO and KEGG pathways, was performed using Metascape online tools. As illustrated in Figures 3B,C, DEGs were mostly enriched in immune response-related terms such as lymphocyte activation (GO:0046649) and natural killer cell mediated cytotoxicity (hsa04650). Finally, a PPI network of 110 DEGs was established and two network modules including nine genes were identified using Metascape (Figure 3D).

### Evaluation of the Modular Genes Using Breast Cancer Cohorts

**FIGURE 4:**
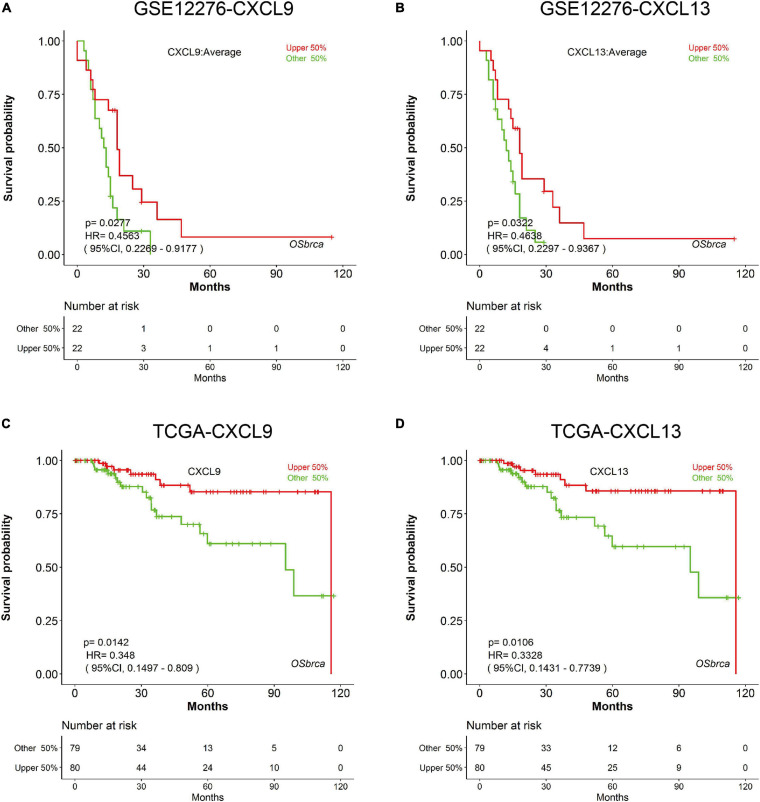
Prognostic validation of CXCL9 and CXCL13 in independent cohorts. K-M analysis suggested that patients with elevated CXCL9 and CXCL13 levels significantly correlated with better OS in **(A,B)** GSE12276 and **(C,D)** TCGA.

In order to evaluate the nine genes from the modules determined by the METABRIC analysis, we used additional TNBC cases obtained from an independent dataset (GSE12276). Survival analyses were performed grouped by the expression level of the nine genes, respectively. Survival curves suggested that patients with elevated CXCL9 and CXCL13 levels experienced significantly improved OS (CXCL9 *P* = 0.0277; CXCL13 *P* = 0.0322; Figures 4A,B). The prognostic roles of CXCL9 and CXCL13 are consistent in the TCGA dataset (CXCL9 *P* = 0.0142; CXCL13 *P* = 0.0106; Figures 4C,D).

**FIGURE 5:**
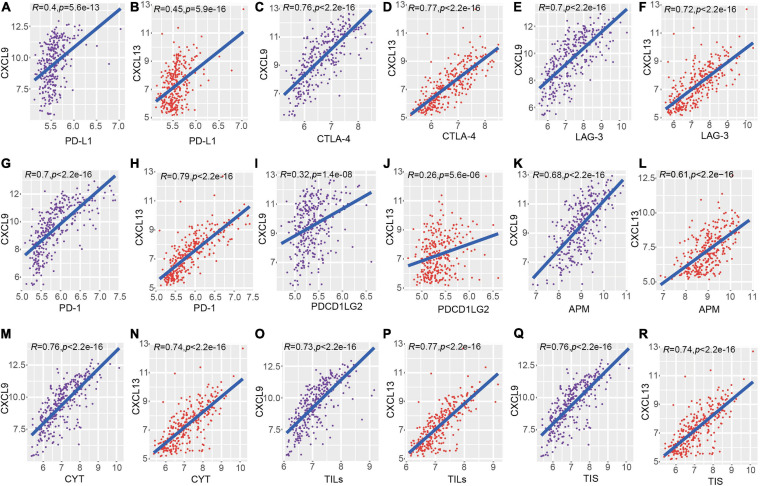
Correlation of CXCL9 and CXCL13 with local immune features and checkpoints across METABRIC TNBC samples. **(A–J)** showed the comparison of checkpoints (PD-L1/CTLA4/LAG3/PD-1/PD-L2) and the **(K–R)** showed the comparison of immune features.

To identify the roles of CXCL9 and CXCL13 across dominant determinants of immune cell infiltration, the association between their expressions and a variety of immune inhibitors (Programmed Cell Death 1, Programmed Cell Death 1 Ligand, Cytotoxic T-Lymphocyte Antigen 4, Lymphocyte Activation protein 3, and programmed cell death 1 ligand 2 (PD-1/PD-L1/CTLA4/LAG3/PD-L2) were analyzed. In the METABRIC dataset, there is a strong correlation of these immune inhibitory genes with both CXCL9 and CXCL13 (Figures 5A–J). Also, some immune tumoral features (such as CYT, APM, TILs, and TIS) were also significantly correlated to the expression level of CXCL9 and CXCL13 (Figures 5K–R). In order to validate the above results, we repeated the same analysis using TNBC samples in the TCGA database. Consistent with the METABRIC dataset, a significant positive correlation of these two genes with immune checkpoints, as well as immune tumoral features, were observed (Supplementary Figure 2).

### CXCL9 and CXCL13 Are Predictive for Therapeutic Response

**FIGURE 6:**
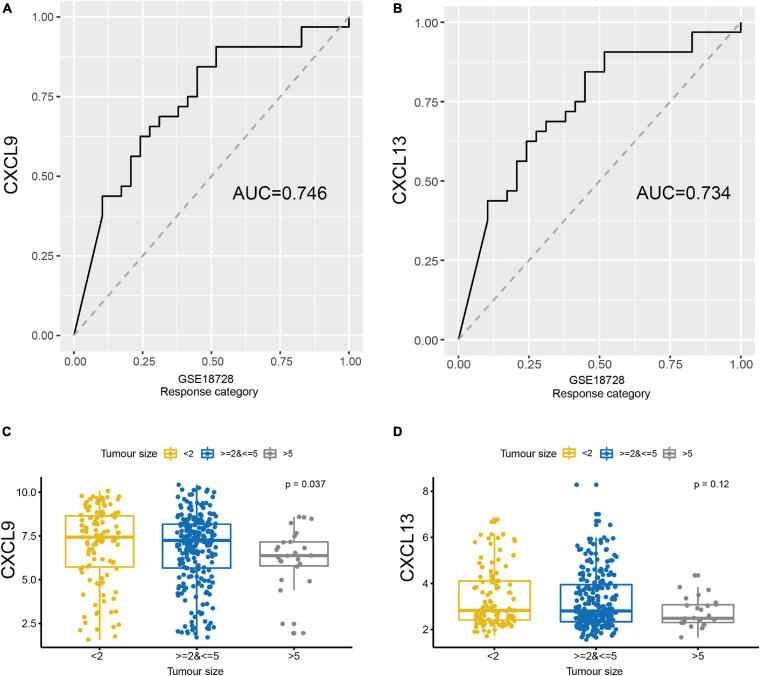
The expression of CXCL9 and CXCL13 could predict the therapeutic benefit. ROC curves were generated to validate the ability of **(A)** CXCL9 and **(B)** CXCL13 to predict chemotherapy response using GSE18728. Comparison of the expression of **(C)** CXCL9 **(D)** and CXCL13 in GSE137356 by the tumor size after chemotherapy.

Two groups of breast cancer datasets, GSE137356 and GSE18728, were used to determine if CXCL9 and CXCL13 expressions could predict chemotherapy response. In the GSE18728 dataset, correlation between baseline gene expression and tumor response to treatment by docetaxel and capecitabine neoadjuvant chemotherapy were assessed (Korde et al., 2010). We used CXCL9 and CXCL13 to predict patients’ response to chemotherapy. The area under the curves (AUC) of the CXCL9 and CXCL13 were 0.746 and 0.734, respectively (Figures 6A,B). To examine whether expressions of CXCL9 and CXCL13 changed in patients with different chemotherapy response, we obtained gene expression data from GSE137356, in which TNBC patients were treated with adjuvant doxorubicin and cyclophosphamide. Figures 6C,D depicts that those demonstrating better clinical response also possessed significantly raised CXCL9 and CXCL13 levels, suggesting that CXCL9 and CXCL13 could potentially predict a better response to chemotherapy.

## Discussion

Amongst the latest to be researched of the multitude of factors known to affect TNBC growth is the degree of tumor infiltration by immune cells. Next-generation sequencing represents useful tools that can be used to meticulously scrutinize the tumor microenvironment. Previous researchers have established profiles of the types of cells occupying the TNBC microenvironment (Burstein et al., 2015; Bonsang-Kitzis et al., 2016; He et al., 2018; Romero-Cordoba et al., 2019; Xiao et al., 2019). However, few studies identified potential prognostic biomarkers from this pool or have further investigated the relationships between chemotherapy response and immune heterogeneity of TNBC.

In the current investigation, well-characterized TNBC datasets were utilized to determine the heterogeneity of the infiltrating cell subpopulation. Our study revealed the existence of two phenotypically distinct TNBC microenvironments and their clinical significance. We found that those of the high infiltration cluster have a higher proportion of majority immune cells, such as CD8+, CD4+, and B cells, as well as macrophages, which usually act as the main initiator of immune responses against the primary tumor (Figure 1A). Conversely, the low infiltration subtype had a relatively lower abundance of immune-active cells (Figures 1B–E). We obtained a similar result using the CIBERSORTX method (Supplementary Figure 3). The ESTIMATE algorithm which calculated immune/stromal/ESTIMATE scores was also used and significant differences between two clusters were observed (Supplementary Figure 2). ESTIMATE is a well-established algorithm used to performed prognostic assessments and exploration of genetic alterations in many neoplasms (Xu et al., 2019). CYT is an important marker of tumor inflammation that is indicative of a microenvironment rich in T cells, thereby imparting effective anti-tumor immunity (Wang et al., 2019). Higher expressions of CYT were predicted to improve prognosis (Senbabaoglu et al., 2016). Our result showed that the high infiltration cluster had higher expressions of CYT (Supplementary Figure 2D). TNBC survival has been linked to TIL levels (Loi et al., 2014; Romero-Cordoba et al., 2019). Thus, TILs have been considered to be able to predict TNBC response to chemotherapy. We found that APM, TILs, and TIS were also found to be at higher levels in the high infiltration cluster (Supplementary Figures 2E–G).

Given the distinct tumor immunophenotypes and their impact on chemotherapy response, we further investigated their clinical implications. Those of the high infiltration cluster had significantly better overall survival than the other two clusters (Figure 2A). These findings reflect prior investigations that have concluded that better clinical outcomes were noted in TNBC with higher activity of immune cells (He et al., 2018; Romero-Cordoba et al., 2019; Xiao et al., 2019; Wang et al., 2020). Clinical prognosis was improved when a higher amount of immune cell infiltrate was present in the tumor (Figure 2B).

We then explored if distinct intrinsic tumor microenvironments were present across TNBC tumors. Other studies have suggested the use of “hot” and “cold” classification of tumor cells (Galon and Bruni, 2019). The latter is used to indicate non-inflamed tumors while the former suggests inflamed T-cell rich tumors (Galon and Bruni, 2019). This pattern of immune classification was also observed in our study. The high infiltration cluster was marked by a higher degree of cytotoxic and non-cytotoxic T cell infiltration, better prognosis, and other immune factors which were considered to be characteristic of “hot” tumors. Conversely, cold tumors have been described to be immunologically quiescent. We also investigate the T-cell function in TCGA dataset. We found that both CD4 + and CD8 + T cell did not show a significant correlation with the overall survival (Supplementary Figures 4A,B), but higher PD1 expression is negatively correlated to OS (Supplementary Figure 4C). Higher and sustained expression of inhibitory multiple inhibitory receptors, such as PD1, is a hallmark of exhausted T cells (Canale et al., 2018).

In order to identify markers with potential prognostic value associated with TME, DEGs between high and low infiltration clusters were analyzed. A total of 110 DEGs were identified (Figure 3A). Functional annotation revealed that these DEGs were predominately enriched in processes involved in activation and regulation of innate immune signaling such as lymphocyte activation (GO:0046649) and natural killer cell mediated cytotoxicity (hsa04650). Modular analysis of Metascape identified two modules including nine genes. We perform multivariate Cox regression analysis on module 1, which included six genes. Using the METABRIC dataset, we identified risk score using the following formula: [Expression level of CCL19^∗^(0.021529)] + [Expression level of CCL5^∗^ (−0.01437)] + [Expression level of CXCL9^∗^ (0.065614)] + [Expression level of CXCL12 ^∗^ (−0.10719)] + [Expressionlevel of CXCL13^∗^ (0.05269)] + [Expression level of C3^∗^ (−0.14915)]. Supplementary Figure 5A shows the comparisons of survival differences between the two groups in the training set (METABRIC; *P* = 0.047). Moreover, such findings were further verified in the testing set (Supplementary Figures 5B–D corresponding to GSE25055, GSE103091, and GSE58812, respectively).

We next evaluated the relationship between OS and the above nine genes using univariate Cox regression. Survival analysis using an independent verification set indicated that two (CXCL9 and CXCL13) of the nine genes were significantly related to TNBC prognosis. Nodal biomarkers (also known as single molecular biomarkers) are sensitive molecules specific to their respective disease that exist in isolation (Lin et al., 2019). To determine if genomic variables were correlated with immune cell infiltrate profiles, levels of macrophages, B cells, CD8^+^, and CD4^+^ T cells between high and low expression group of CXCL9 and CXCL13 were evaluated. We found that the expression of CXCL9 and CXCL13 were positively correlated to the immune cell infiltrates (Supplementary Figures 6A–H). To further evaluate the role of CXCL9 and CXCL13 in tumor immunity, we performed comparisons between the above two genes and immune markers (CYT, APM, TILs, and TIS), as well as checkpoint expression, and found that a statistically significant correlation existed between them in both the METABRIC and TCGA dataset, suggesting that a higher expression of CXCL9 and CXCL13 were associated with enhanced immune cell infiltrate.

As an IFN-γ inducible chemokine, CXCL9 is a significant mediator of interaction between the host and tumor. Raised levels of CXCL9 correlated with higher amounts of tumor-infiltrating natural killer (NK) cells and longer postoperative survival (Fukuda et al., 2020). CXCL13 is secreted by TFH cells, and is involved in a positive feedback mechanism that enhances levels of memory, cytotoxic, T helper 1 (TH1), and TFH T cells, as well as B cells, in breast cancer (Gu-Trantien et al., 2017).

We further investigate the relationship between TILs and clinical outcome. We found that high TILs cluster have markedly better overall survival (OS) in contrast to the low cluster (Supplementary Figure 7A). We also found that the “TILs-low and CXCL9/13-low” class had the worst survival, and the other three groups did not display a significant difference (Supplementary Figures 7B,C). Interestingly, although higher levels of TILs and CXCL9 were protective factors of TNBC, the “TILs-high and CXCL9/13-high” did not display a significantly better survival (Supplementary Figures 7B,C), suggesting that there may be an intricate interaction in the tumor microenvironment. Several studies have shown that CXCL9 had both positive and negative effects; on the one hand, overexpression of CXCL9 has shown to reduce tumor progression by inhibiting angiogenesis. On the other hand, CXCL9 can act directly on tumor cells expressing the CXCR3 receptor to promote cell migration and epithelial mesenchymal transition (Neo and Lundqvist, 2020). It is still necessary to explore CXCL9-induced signaling cascade via CXCR3 in CD8^+^ T cells.

The primary modality of treatment in breast cancer is chemotherapy, an agent that is strongly affected by the immune microenvironment. Published reports indicate that breast cancer patients with higher TILs demonstrate a more complete pathological response post- neoadjuvant chemotherapy (Loi et al., 2014). To evaluate the chemotherapy-response upon CXCL9 and CXCL13, two breast cancer cohorts that possessed information regarding chemotherapy response were extracted from the GEO dataset. We found that CXCL9 and CXCL13 were good predictors of chemotherapy response and their expressions were higher in patients who responded well to chemotherapy than the non-responsive ones (Figure 6).

In summary, we discerned two distinct TNBC subtypes (high or low immunity) and identified two markers associated with prognosis and chemotherapy response. This data further enhances the concept and supports the clinical importance of TNBC immune heterogeneity. However, there are still some limitations in our study. More sufficient datasets are needed to validate the immune signatures and more experimental research is necessary to fully elucidate the biological or medical mechanisms underlying immune heterogeneity.

## Data Availability Statement

The original contributions presented in the study are included in the article/Supplementary Material, further inquiries can be directed to the corresponding author/s.

## Author Contributions

YL and YZ designed the study. DL collected data. DL and XL developed the computational model and analyzed the network. YL, PX, and JZ wrote the manuscript. All authors reviewed the manuscript.

## Conflict of Interest

The authors declare that the research was conducted in the absence of any commercial or financial relationships that could be construed as a potential conflict of interest.

## Footnotes

1 https://cibersortx.stanford.edu/

2 http://metascape.org

3 http://string-db.org

## Supplementary Material

Supplementary Figure 1 The comparison of ESTIMATE results and immune features between two microenvironment clusters in the TNBC. **(A)** ESTIMATE scores. **(B)** Immune score. **(C)** Stromal score. **(D–G)** Boxplots showed the difference of **(D)** CYT, **(E)** APM, **(F)** TILs, and **(G)** TIS.

Supplementary Figure 2 Correlation of CXCL9 and CXCL13 with local immune features and checkpoints in TCGA TNBC samples. **(A–J)** showed the comparison of checkpoints (PD-L1/CTLA4/LAG3/PD-1/PD-L2) and **(K–R)** showed the comparison of immune features.

Supplementary Figure 3 Cell abundances of **(A)** CD8 T cell, **(B)** CD4 T cell, **(C)** B cell, and **(D)** Macrophage estimated by CIBERSORTx.

Supplementary Figure 4 Prognostic validation of T cells. Both CD4 + **(A)** and CD8 + T cell **(B)** did not show a significant correlation with the overall survival, but PD1 expression is negatively correlated to OS **(C)**.

Supplementary Figure 5 Multivariate Cox regression analysis of 6 genes in module 1. Survival curves of patients in high-risk group and low-risk group of the training set **(A)**, the testing set **(B)** GSE25055, **(C)** GSE103091, and **(D)** GSE58812 are shown.

Supplementary Figure 6 The relationship between gene expression and microenvironment cytotypes. Levels of macrophages, B cells, CD8 +, and CD4 + T cells between high and low expression group of CXCL9 **(A–D)** and CXCL13 **(C–H)** were evaluated.

Supplementary Figure 7 Kaplan–Meier curves for immune infiltration -specific survival based on TILs and CXCL9/13 expression classes. The K-M curves for **(A)** TILs **(B)** TILs and CXCL9 and **(C)** TILs and CXCL13.
