# Supplementary figures and images for: Immune Cell Infiltration-Based Characterization of Triple-Negative Breast Cancer Predicts Prognosis and Chemotherapy Response Markers
Source: Front Genet. 2021 Mar 19;12:616469. doi: 10.3389/fgene.2021.616469 (PMC8017297; doi:10.3389/fgene.2021.616469)

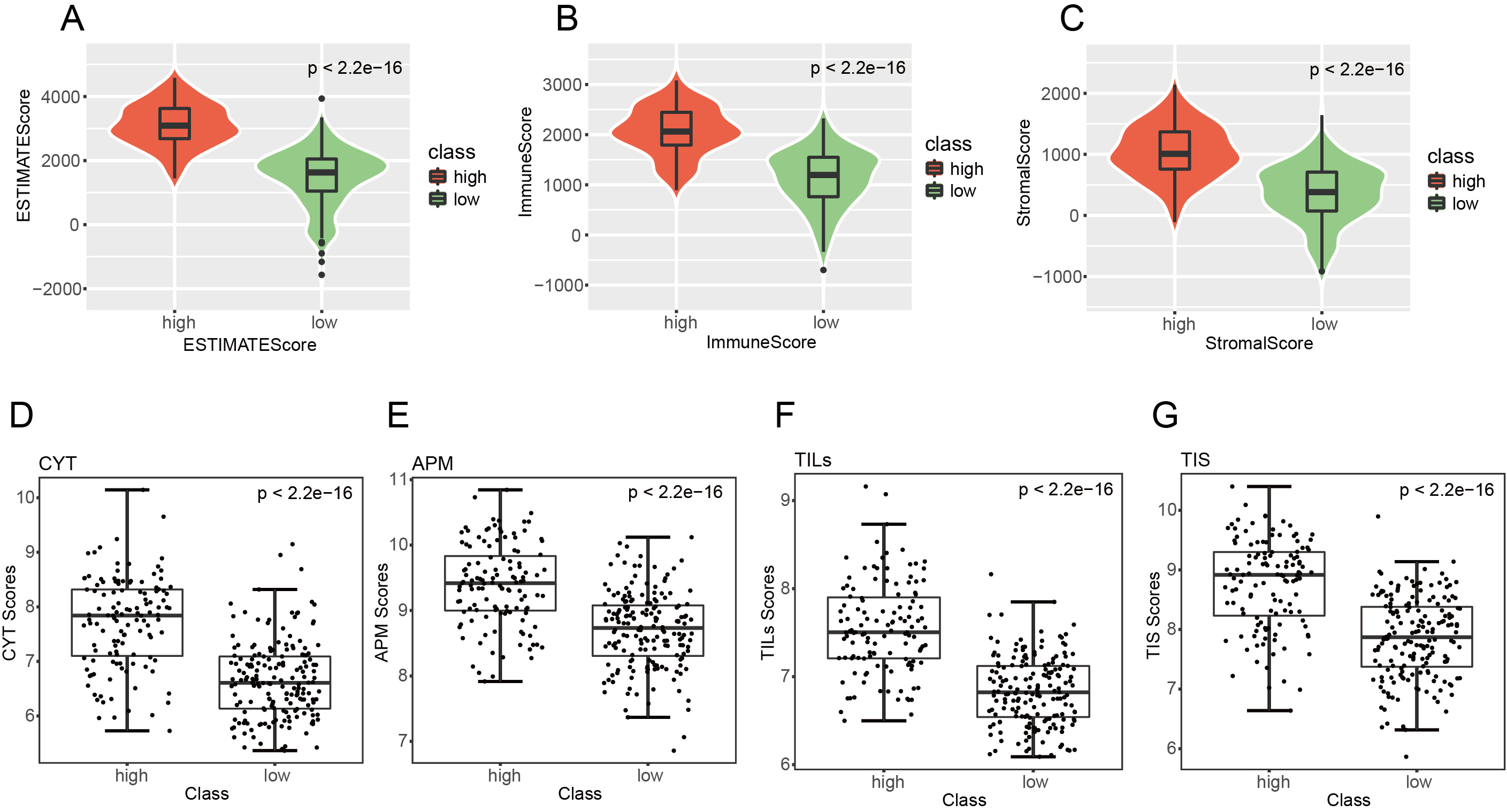

Supplement: Supplementary Figure 1 — The comparison of ESTIMATE results and immune features between two microenvironment clusters in the TNBC. (A) ESTIMATE scores. (B) Immune score. (C) Stromal score. (D–G) Boxplots showed the difference of (D) CYT, (E) APM, (F) TILs, and (G) TIS. [file Image_1.JPEG]

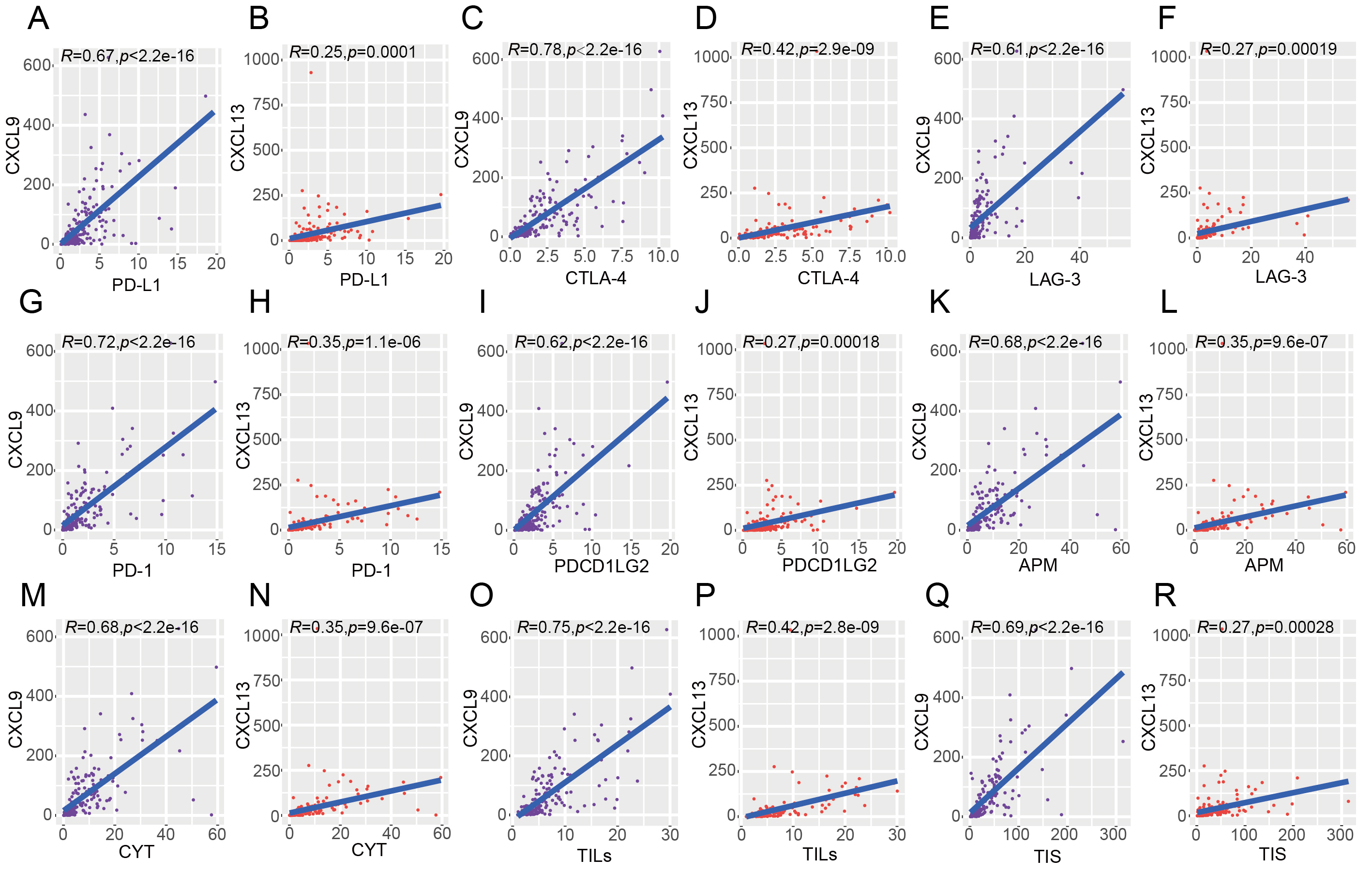

Supplement: Supplementary Figure 2 — Correlation of CXCL9 and CXCL13 with local immune features and checkpoints in TCGA TNBC samples. (A–J) showed the comparison of checkpoints (PD-L1/CTLA4/LAG3/PD-1/PD-L2) and (K–R) showed the comparison of immune features. [file Image_2.JPEG]

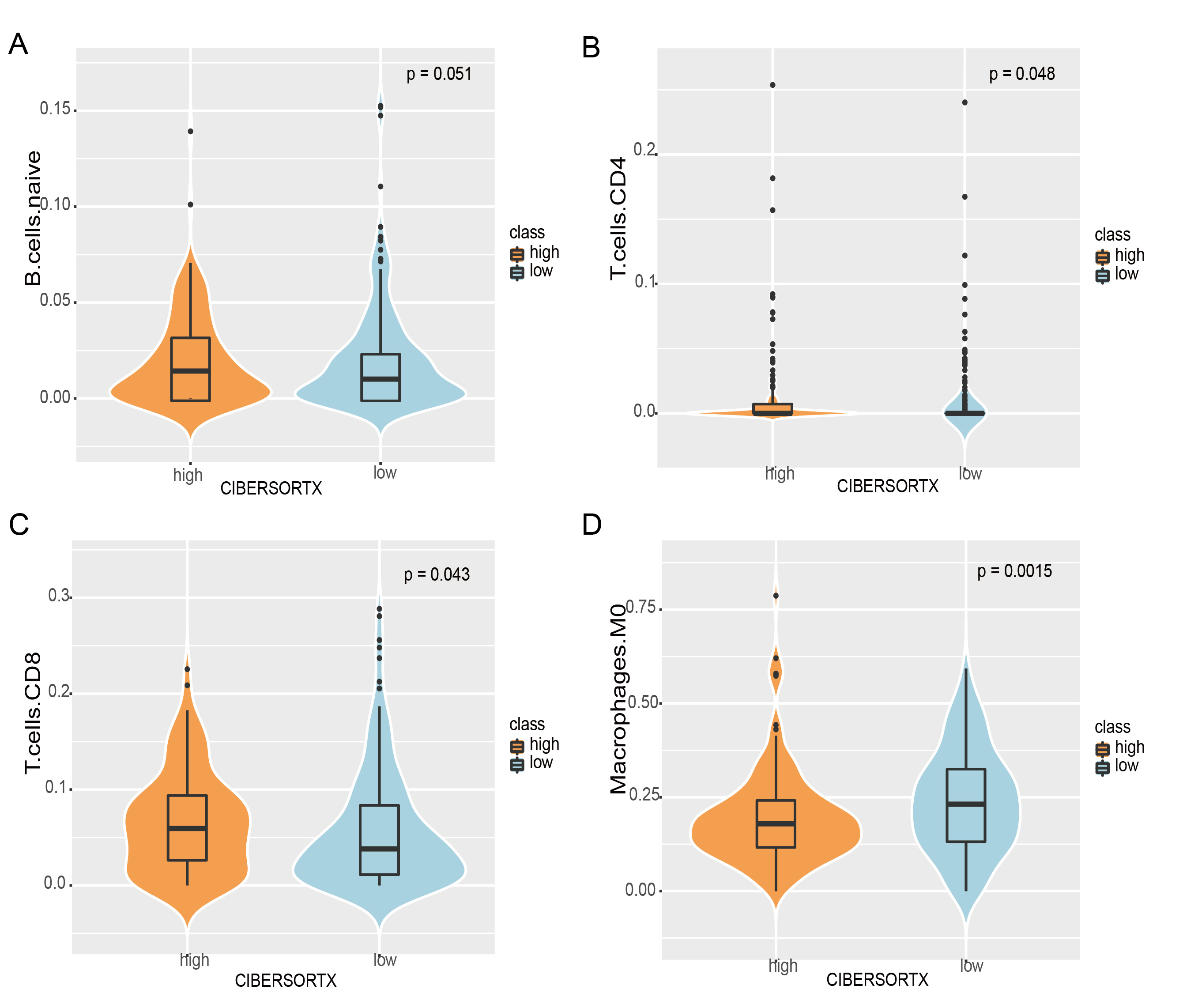

Supplement: Supplementary Figure 3 — Cell abundances of (A) CD8 T cell, (B) CD4 T cell, (C) B cell, and (D) Macrophage estimated by CIBERSORTx. [file Image_3.JPEG]

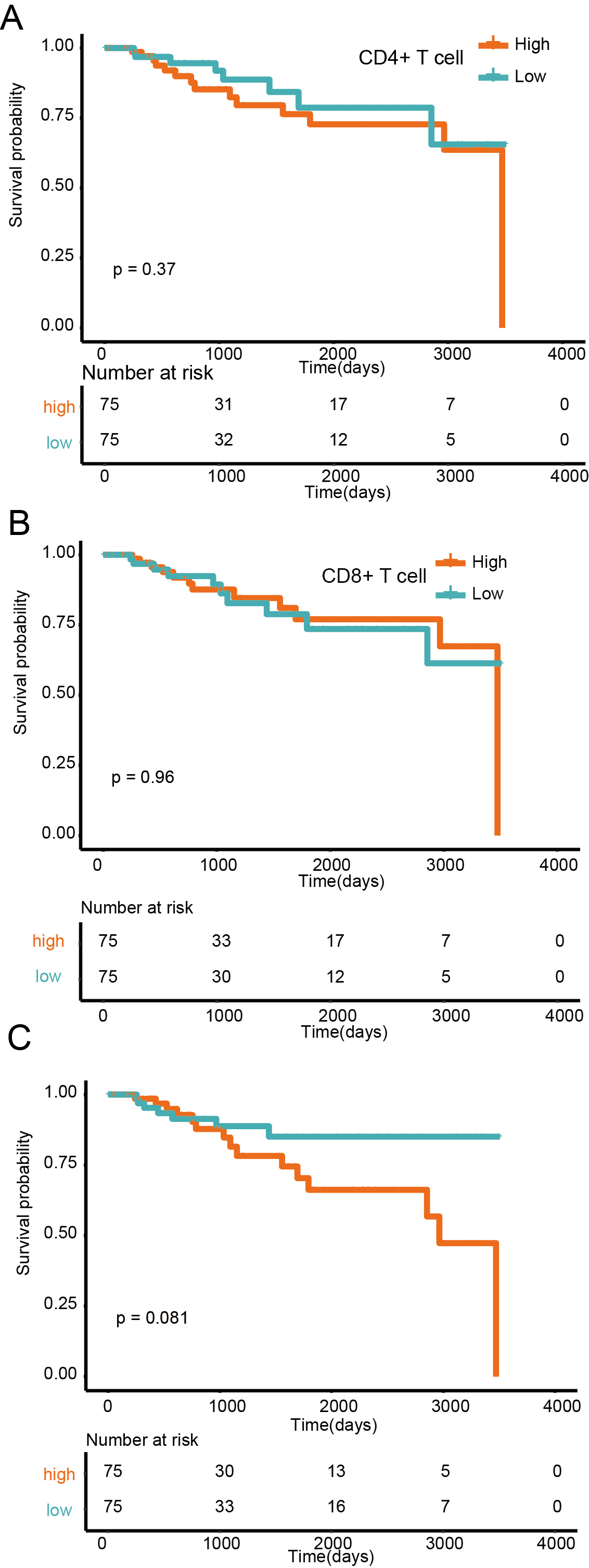

Supplement: Supplementary Figure 4 — Prognostic validation of T cells. Both CD4 + (A) and CD8 + T cell (B) did not show a significant correlation with the overall survival, but PD1 expression is negatively correlated to OS (C). [file Image_4.JPEG]

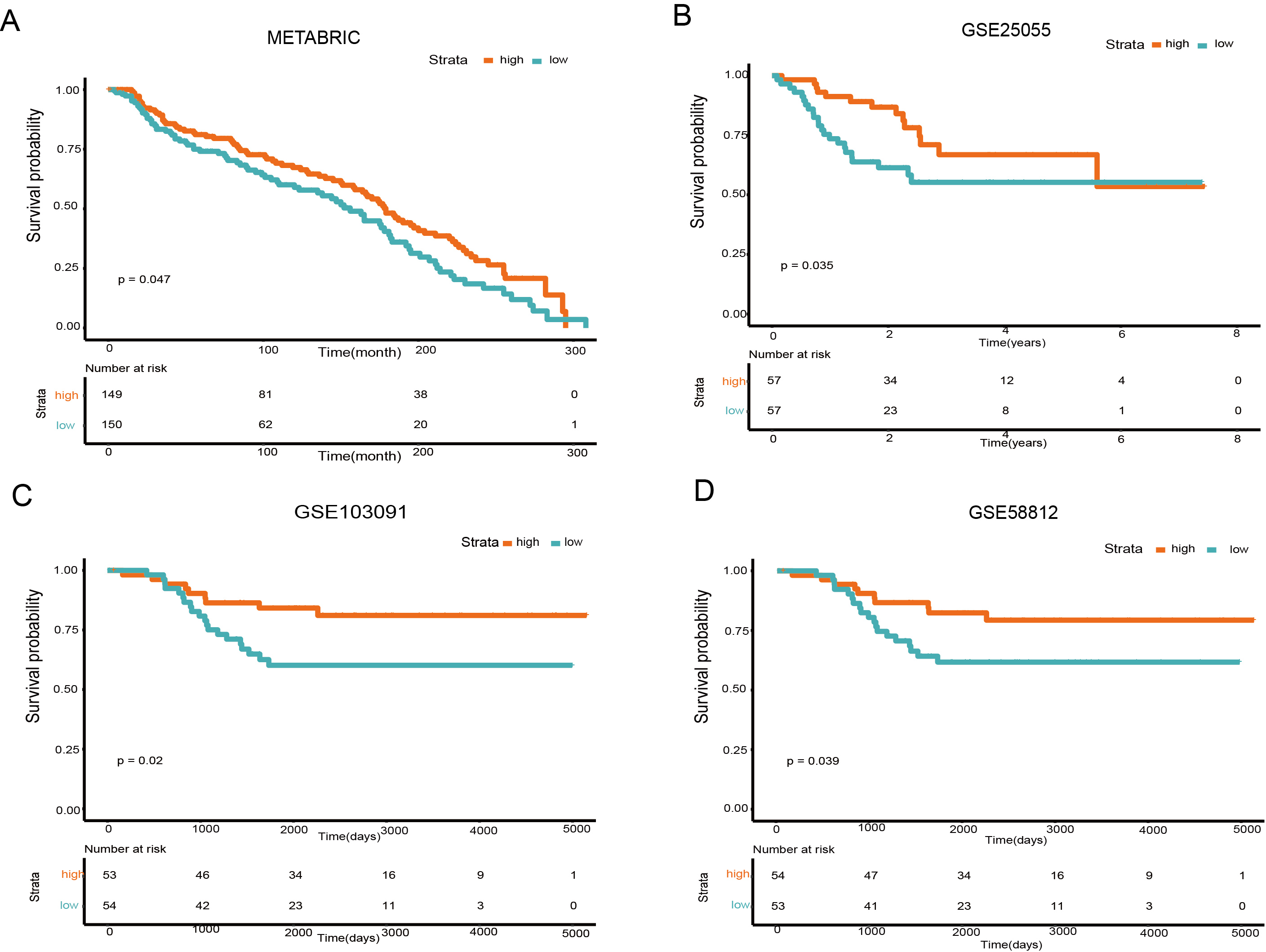

Supplement: Supplementary Figure 5 — Multivariate Cox regression analysis of 6 genes in module 1. Survival curves of patients in high-risk group and low-risk group of the training set (A), the testing set (B) GSE25055, (C) GSE103091, and (D) GSE58812 are shown. [file Image_5.JPEG]

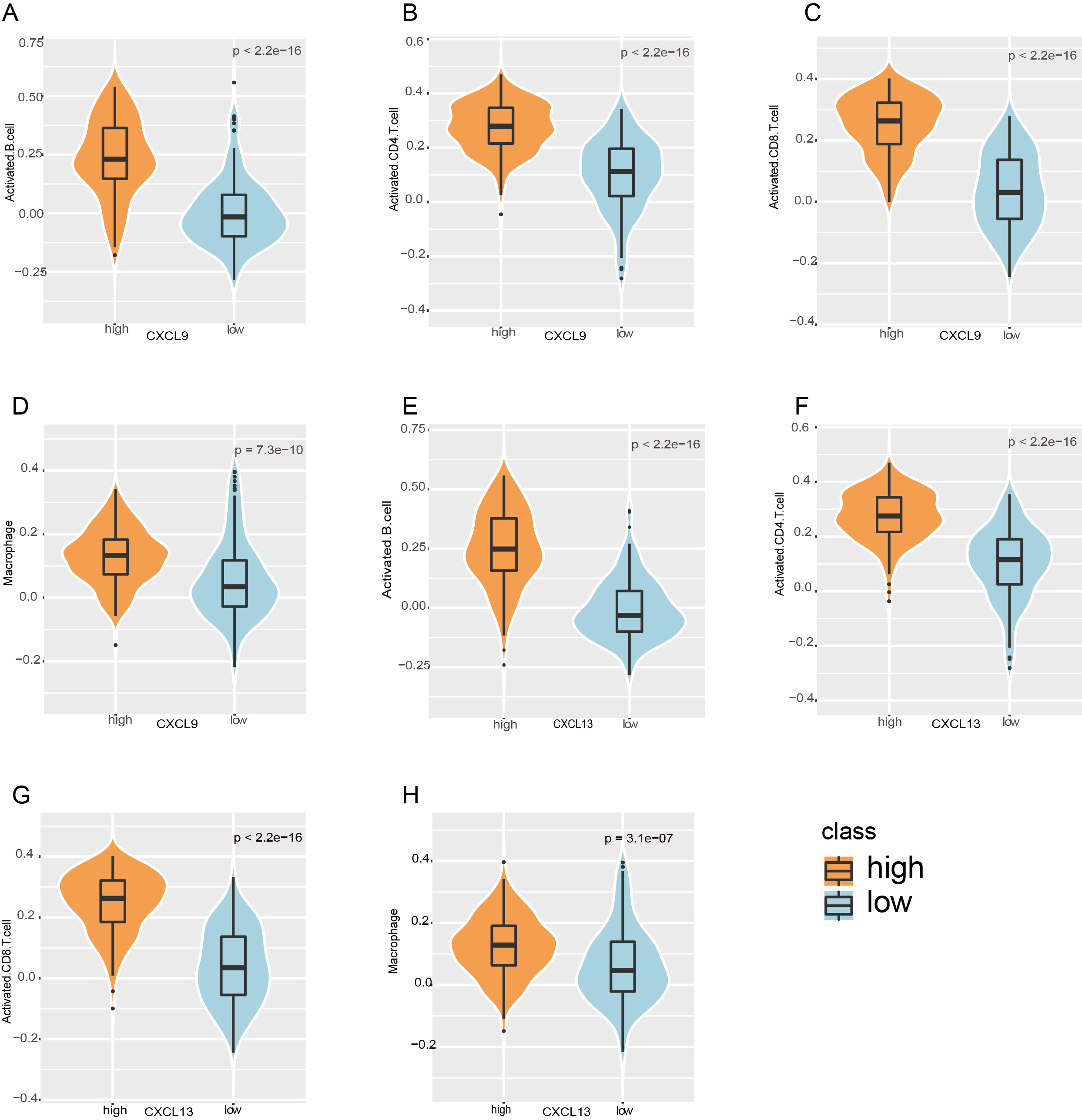

Supplement: Supplementary Figure 6 — The relationship between gene expression and microenvironment cytotypes. Levels of macrophages, B cells, CD8 +, and CD4 + T cells between high and low expression group of CXCL9 (A–D) and CXCL13 (C–H) were evaluated. [file Image_6.JPEG]

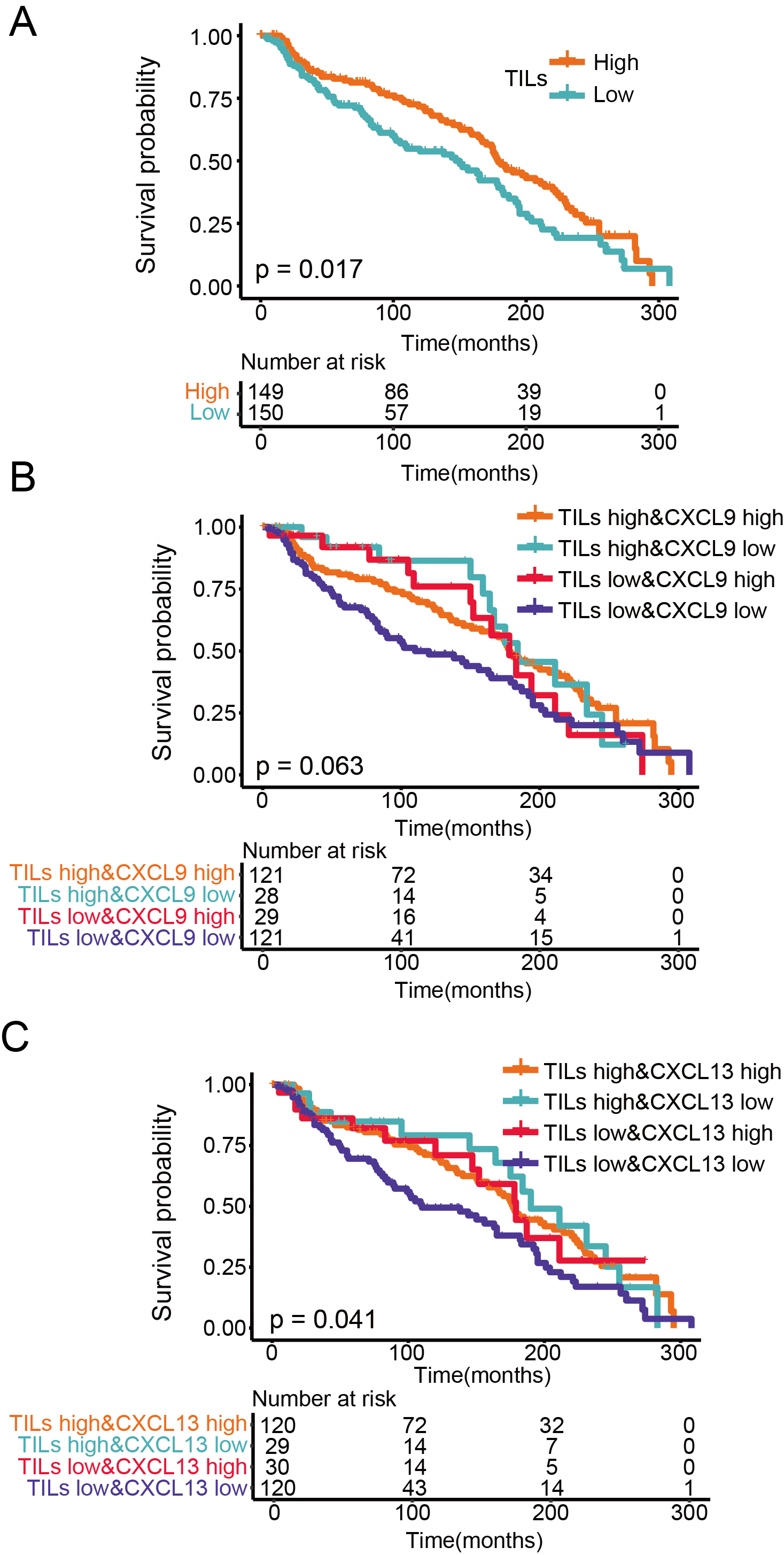

Supplement: Supplementary Figure 7 — Kaplan–Meier curves for immune infiltration -specific survival based on TILs and CXCL9/13 expression classes. The K-M curves for (A) TILs (B) TILs and CXCL9 and (C) TILs and CXCL13. [file Image_7.JPEG]
